# Geographical origin of *Plasmodium vivax *in the Republic of Korea: haplotype network analysis based on the parasite's mitochondrial genome

**DOI:** 10.1186/1475-2875-9-184

**Published:** 2010-06-25

**Authors:** Moritoshi Iwagami, Seung-Young Hwang, Megumi Fukumoto, Toshiyuki Hayakawa, Kazuyuki Tanabe, So-Hee Kim, Weon-Gyu Kho, Shigeyuki Kano

**Affiliations:** 1 Department of Tropical Medicine and Malaria, Research Institute, National Center for Global Health and Medicine, 1-21-1 Toyama, Shinjuku, Tokyo 162-8665, Japan; 2 Department of Parasitology, Inje University, College of Medicine, 633-165 Gaegum-dong, Busanjin-gu, Busan 614-735, Korea; 3 Graduate School of Comprehensive Human Sciences, University of Tsukuba, 1-1-1 Tennodai, Tsukuba, Ibaraki 305-8577, Japan; 4 Laboratory of Malariology, Research Institute for Microbial Diseases, Osaka University, Suita, Osaka 565-0871, Japan; 5 Department of Malariology, Paik Institute of Clinical Research, Inje University, College of Medicine, 633-165 Gaegum-dong, Busanjin-gu, Busan 614-735, Korea

## Abstract

**Background:**

The Republic of Korea (South Korea) is one of the countries where vivax malaria had been successfully eradicated by the late 1970s. However, re-emergence of vivax malaria in South Korea was reported in 1993. Several epidemiological studies and some genetic studies using antigenic molecules of *Plasmodium vivax *in the country have been reported, but the evolutionary history of *P. vivax *has not been fully understood. In this study, the origin of the South Korean *P. vivax *population was estimated by molecular phylogeographic analysis.

**Methods:**

A haplotype network analysis based on *P. vivax *mitochondrial (mt) DNA sequences was conducted on 11 *P. vivax *isolates from South Korea and another 282 *P. vivax *isolates collected worldwide.

**Results:**

The network analysis of *P. vivax *mtDNA sequences showed that the coexistence of two different groups (A and B) in South Korea. Groups A and B were identical or close to two different populations in southern China.

**Conclusions:**

Although the direct introduction of the two *P. vivax *populations in South Korea were thought to have been from North Korea, the results of this analysis suggest the genealogical origin to be the two different populations in southern China.

## Background

Malaria is distributed not only in tropical and subtropical areas but also in some temperate areas of the world. *Plasmodium falciparum*, which is distributed in tropical and subtropical areas, accounts for 90% of malaria cases. Like *P. falciparum, Plasmodium vivax *is distributed in tropical and subtropical areas, but its range extends to some temperate areas. In Asian and South American countries, the proportion of *P. falciparum *cases is gradually decreasing due to global malaria controls programmes, such as The Roll Back Malaria Partnership and The Global Fund. On the other hand, the proportion of *P. vivax *cases is gradually increasing [1]. Therefore, *P. vivax *should be given greater attention than it has received.

The Republic of Korea (South Korea) is one of the countries where vivax malaria had been successfully eradicated by the late 1970s. This was due to an effective national eradication programme conducted by the National Malaria Eradication Service under the operation of the South Korean government with the support of the WHO [2-4]. However, in 1993, the first case of indigenous vivax malaria after the eradication program was reported from the border area between North and South Korea in the western Demilitarized Zone (DMZ) [5]. The number of cases steadily increased until 2000 (4,142 cases), at which point they began to gradually decrease until 2004 (864 cases). However, in 2005, 2006 and 2007, the number of reported cases increased again (1,311, 2,019 and 2,203 cases, respectively) [6]. Initially, the patients were South Korean soldiers or veterans that had served in the western DMZ. However, the numbers of vivax cases among civilians living in the area were also gradually increasing [6]. According to the WHO, vivax malaria in the Democratic People's Republic of Korea (North Korea), with 99,582 reported cases in 1999; 298,058 cases in 2001; and 34,485 cases in 2004, was more prevalent than in South Korea [7,8].

*Plasmodium vivax *in South and North Korea has unique characteristics, such as a long incubation period (maximum 13 months), seasonal transmission (only the summer season) and it is adapted to a cold climate [3,9-15]: the endemic areas are covered with snow in winter season. Although the evolutionary history of *P. vivax *from other countries has recently been addressed, thus far the history of *P. vivax *in the Korean peninsula (South and North Korea) has not been clearly understood [16-18]. Several epidemiological data showed that the re-emergence of vivax malaria in South Korea would be the introduction from North Korea through the DMZ [3-8]. However, the geographical origin of *P. vivax *population in the Korean peninsula has not been determined so far. In the present study, in order to estimate geographical origin of the *P. vivax *population in the Korean peninsula, phylogeographic analysis of the *P. vivax *population in South Korea and the other populations worldwide (including a North Korean isolate) was conducted.

## Materials

### Sample collection

Ten blood samples were collected from vivax malaria patients who were South Korean soldiers that served in the DMZ in 1999. One blood sample was collected from a Korean visitor to Japan in 2002. He was a veteran in the Korean army who had served in the DMZ before he visited Japan and had never been abroad before his visit [19,20]. The patient blood samples were preserved at -30°C until use. These patients were also diagnosed by microscopic examination of peripheral blood smears. This study was performed according to the ethical guidelines for epidemiological studies provided by the Ministry of Education, Culture, Sports, Science and Technology and the Ministry of Health, Labour and Welfare of Japan.

### DNA extraction, Polymerase chain reaction (PCR) and DNA sequencing

The parasite DNA was extracted from the frozen whole blood samples by phenol-chloroform extraction after proteinase K digestion [21]. The whole mitochondrial (mt) DNA sequences (approx. 6 Kb) of the *P. vivax *isolates form South Korea were amplified by PCR using three pairs of primer sets:

Pv-mt1 F (5'-TTCCACTACCAAAATATAATCTCCT-3')

Pv-mt1 R (5'-CACACAAAATCACCGTTCTTATAAA-3')

Pv-mt2 F (5'-TAAATGTGCTTTAATATTATTATAG-3')

Pv-mt2 R (5'-CATAATTCCATAAGAAATTAATATT-3')

Pv-mt3 F (5'-ATCAACAATGACTTTATTTGGTTTA-3')

Pv-mt3 R (5'-ACTATAAAACATGTGATCTAATTAC-3'), which were designed form the mt sequence of the *P. vivax *af20012 isolate [GenBank: AY791517]. Three amplified DNA fragments (approx. 2 Kb) overlapped with each other. Sequencing of the PCR products was performed using an Applied Biosystems 310 Genetic Analyzer (Applied Biosystems Inc, Foster City, CA, USA), using ABI PRISM Big Dye Terminator v.3.1 (Applied Biosystems Inc, Foster City, CA, USA).

### Phylogenetic analysis

MtDNA sequences (approx. 6 Kb) of the 11 *P. vivax *isolates from South Korea (present study) [DDBJ: AB550270-AB550280] and another 282 *P. vivax *isolates collected worldwide that had been deposited in the GenBank database, were used for phylogenetic analysis [16,17]. Mu *et al *[16] deposited 176 sequences [GenBank: AY791517.1-AY791692.1]. Jongwutiwes *et al *[17] deposited 106 sequences [GenBank: AY598035.1-AY598140.1]. DNA alignment of the whole mtDNA sequences of the *P. vivax *isolates was performed by the DNA Alignment version 1.3.0.1 computer software (Fluxus Technology Ltd.) [22]. A haplotype network was constructed based on polymorphic sites of the whole mtDNA sequences of the isolates using the Median-Joining method in the NETWORK version 4.5.1.6 computer software (Fluxus Technology Ltd.) [23].

## Results and Discussion

**Figure 1:**
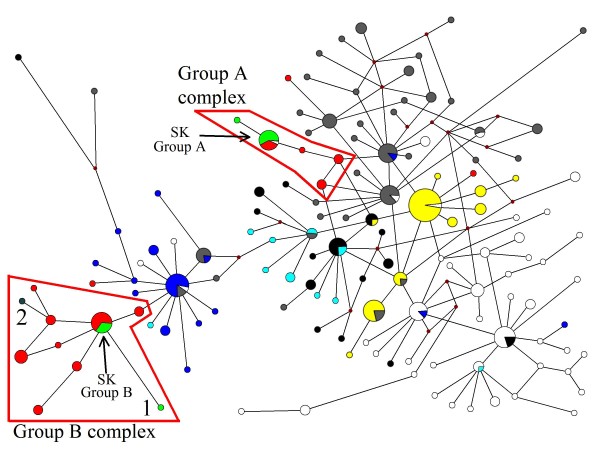
**A haplotype network using mitochondrial genome of *P. vivax *populations**. A haplotype network inferred by a median-joining method, using the 293 mitochondrial (mt) DNA sequences of *P. vivax *populations of which 11 are South Korean isolates (present study) and the other 282 are worldwide isolates that were deposited the GenBank database [16-18]. The size of the each circle represents the frequencies of the haplotype, with each color showing the geographical origin of the isolates. Green indicates isolates from South Korea (present study) and dark green indicates North Korea (the GenBank database). Red indicates isolates from southern China. Blue indicates isolates from Indonesia. Gray indicates isolates from Thailand, Vietnam and Bangladesh. White indicates isolates from Papua New Guinea, Vanuatu and the Solomon Islands. Black indicates isolates from India, Pakistan and Iran. Yellow indicates isolates from Central and South America. Light blue indicates isolates from Africa. No. 1 and 2 in the group B complex indicate isolates from South Korea (the first imported case into Japan from South Korea) and an isolate from North Korea, respectively.

A haplotype network was inferred by the 282 mtDNA sequences of *P. vivax *populations that had been collected worldwide, together with the 11 South Korean isolates (Figure 1). The network tree indicated that two groups of *P. vivax *populations coexist in South Korea (i.e. SK group A and B). The isolates of the SK group A (shown in green) were genetically identical or close to some isolates from southern China (shown in red), and those of the SK group B (shown in green) were also genetically identical or close to other isolates from southern China (shown in red). A neighbour-joining (NJ) tree (Additional File 1a, b) using the same data set for the network analysis were clearly demonstrated that the isolates of the SK group A were clustered with some isolates from southern China, and those of the SK group B were also clustered with other isolates from southern China. Therefore, the two clusters as defined in the NJ tree were named group A complex and group B complex, respectively. The boundaries of the two group complexes were shown in red boxes.

In a previous report, Kho *et al *[24] also noted the observation of two types of genotypes (i.e. SK type A and B) in some antigenic molecules of *P. vivax *in South Korean populations. In the present study, 91% of the isolates (10/11) were correlated to the results of the previous studies on the antigenic molecules: the South Korean isolates in the group A complex had SK type A antigenic molecules, and the South Korean isolates in the group B complex had SK type B antigenic molecules (Additional File 2).

The group A complex was genetically close to Southeast Asian populations (shown in gray); most of them were isolates from Thailand and Vietnam, or close to the South Asian population (shown in black); they were isolates from India, Pakistan and Iran (Figure 1). The group B complex was genetically close to the Indonesia population (shown in blue). Generally, old (or ancestral) populations are more genetically diverged than young populations. In this context, the Southeast Asian populations (shown in gray) and Papua New Guinean populations (shown in white) seemed old populations. Considering a possible population expansion, the group B complex seemed to have diverged from the Indonesian population (Figure 1). The genetic divergence of the extant *P. vivax *populations in the world is presumably a result of ancient hominid geographic expansion [17]. Therefore, the relationships between the group B complex and the Indonesian population suggests a possibility that the expansion of *P. vivax *population(s) from Indonesia to southern China was brought about with the migration(s) of ancient hominids. Another possible scenario is that the group B complex has directly diverged from the Southeast Asian populations because some isolates from Southeast Asia are identical to those from Indonesia. In this scenario, the expansion of *P. vivax *population(s) from Southeast Asia to Indonesia was also brought about with the migration(s) of ancient hominids. Although the present South Korean *P. vivax *populations are believed to have recently derived from North Korea via the DMZ, this study suggests that the *P. vivax *lineages in the Korean peninsula have their genealogical ancestor in *P. vivax *populations from southern China.

One of the remarkable characteristics of *P. vivax *in Korean peninsula is its evolutionary adaptation to the cold climate. The long incubation period of Korean *P. vivax *is the key to the adaptation, because the parasites in the liver cells of the human host appears in the blood streams from the liver cells mainly between June and September (around the summer season) when mosquitoes are highly prevalent, but the parasites remain in the liver cells in the other colder seasons when mosquitoes are not present [3,9,15]. In this phenomenon, it seems as if the parasites are waiting for the mosquitoes within the host liver cells by regulating the duration of the incubation period.

Several workers reported that there seem to be two types of *P. vivax *strains (or populations) in the Korean peninsula: one with a short incubation period and the other with a long incubation period. The incubation period of the former type of North Korean strain is 14 days to 1 month, and the incubation period of the latter type of strain is 8 months to 13 months, as determined by experimental infection to humans through bites of the infected mosquitoes [25]. The proportion of strains (or populations) showing the short incubation period was 26.0%, whereas the proportion of strains (or populations) showing the long incubation period was 74.0% [25]. One mtDNA sequence of a North Korean isolate deposited in the GenBank database was included in the present study. The North Korean isolate was shown as No. 2 (shown in dark green) in the group B complex in the haplotype network in Figure 1. The information of the incubation period of the North Korean isolate was not obtained.

One isolate from the imported patient in Japan [19,20] with a long incubation period (at least eight months) was also grouped in the group B complex shown as No. 1 in the haplotype network (Figure 1). The information on the duration of the incubation period of the other 10 South Korean isolates used in this study has not been obtained thus far, but the branching patterns in the network tree appear to be related to the phenotypic characteristics of the parasites within the host.

Further study is needed to demonstrate whether the two groups of South Korean isolates (groups A and B) correlate with some clinical or epidemiological differences in the endemic areas. Haplotype network analysis using the mtDNA sequences of *P. vivax *is a useful tool for estimating the geographical origin of isolates as well as for the prediction of probable phenotypes.

## Conclusion

The direct introduction of the present *P. vivax *populations to South Korea is thought to be from North Korea via the DMZ, but the true origin of the *P. vivax *populations in the Korean peninsula is now suggested to be from the two different *P. vivax *populations in southern China.

## Competing interests

The authors declare that they have no competing interests.

## Authors' contributions

MI and SYH carried out the molecular genetic studies, performed the phylogenetic analysis and drafted the manuscript. MF and SHK helped the molecular genetic studies and helped with the writing of the manuscript. TH and KT helped the phylogenetic analysis and helped with the writing of the manuscript. WGK collected the patients' blood samples, participated in the design of the study, acquisition of funding, coordination and writing of the manuscript. SK participated in the design of the study, acquisition of funding, coordination and writing of the manuscript. All authors read and approved the final manuscript.

## Supplementary Material

Additional file 1 **A neighbour-joining (NJ) tree inferred by mitochondrial DNA sequences of *P. vivax***. The NJ tree was constructed by MEGA version 3.1 (Kumar S, Tamura K, Nei M: MEGA3: Integrated Software for Molecular Evolutionary Analysis and Sequence Alignment. *Bioinformatics *2004, **5**:150-163) using Kimura's 2-parameter model for calculating genetic distance. Additional File 1a continued to Additional File 1b. The isolate (AY598125 Vietnam) with Asterisk (*) at the bottom of the Additional File 1a is identical to the isolate with Asterisk at the top of the Additional File 1b. The eleven South Korean isolates (present study) were clustered into either Group A complex or Group B complex in the Additional File 1b. Click here for file

Additional file 2 **Genotype of antigenic molecules and mitochondria of the eleven South Korean *P. vivax *isolates**. PvCSP: *P. vivax *circumsporozoite protein, PvDBP: *P. vivax *Duffy binding protein, PvMSP-1: *P. vivax *merozoite surface protein-1, Conditions of PCR and DNA sequencing for antigenic molecules have been previously reported elsewhere [24]. *Genotype of the antigenic molecules of the isolate South Korea B1 was not consistent with that of the mitochondorial genome. Click here for file
