# Supplementary figures and images for: Geographical origin of Plasmodium vivax in the Republic of Korea: haplotype network analysis based on the parasite's mitochondrial genome
Source: Malar J. 2010 Jun 25;9:184. doi: 10.1186/1475-2875-9-184 (PMC2908639; doi:10.1186/1475-2875-9-184)

## Slide 1
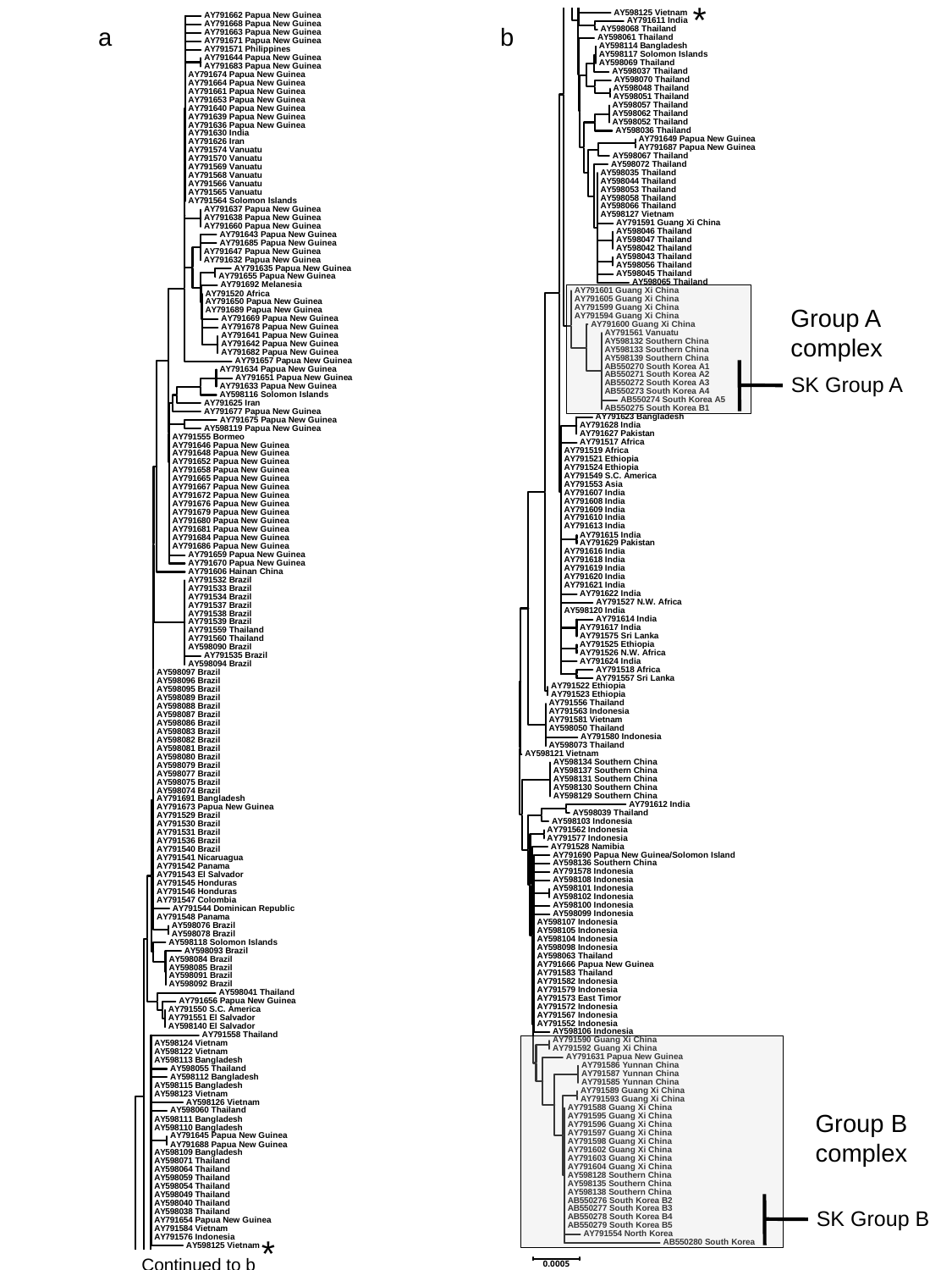

*
a
b
Group A complex
SK Group A
Group B complex
SK Group B
*
Continued to b

Supplement: Additional file 1 — A neighbour-joining (NJ) tree inferred by mitochondrial DNA sequences of P. vivax. The NJ tree was constructed by MEGA version 3.1 (Kumar S, Tamura K, Nei M: MEGA3: Integrated Software for Molecular Evolutionary Analysis and Sequence Alignment. Bioinformatics 2004, 5:150-163) using Kimura's 2-parameter model for calculating genetic distance. Additional File 1a continued to Additional File 1b. The isolate (AY598125 Vietnam) with Asterisk (*) at the bottom of the Additional File 1a is identical to the isolate with Asterisk at the top of the Additional File 1b. The eleven South Korean isolates (present study) were clustered into either Group A complex or Group B complex in the Additional File 1b. [file 1475-2875-9-184-S1.PPT]
